# Photoacoustic spectrum analysis for spherical target size and optical property determination: A feasibility study

**DOI:** 10.1016/j.pacs.2023.100534

**Published:** 2023-07-25

**Authors:** M. Dantuma, D.B. Gasteau, S. Manohar

**Affiliations:** Multi-Modality Medical Imaging Group (M3I), TechMed Centre, University of Twente, Enschede, The Netherlands

**Keywords:** Photoacoustics, Spheres, Frequency spectrum analysis, Feasibility study

## Abstract

The photoacoustic signal generated by an optically absorbing target is determined by the spatial profile of absorbed optical energy within the target. The analysis of the time profile and frequency content of the signal enables the recovery of the geometry of the object, as well as information about the optical properties. The photoacoustic response of spheres with a homogeneous absorbed optical energy profile is well described, and it is known that the width of the photoacoustic pulse is determined by the diameter of the sphere and its sound speed. In practice, the optical attenuation coefficients within the sphere will result in an inwardly decaying fluence profile leading to a similarly decaying absorbed optical energy profile. Further, the optical attenuation coefficients may be inhomogeneously distributed in the sphere. The implication for both cases is that the existing model for spheres does not fully apply. In this work, we developed analytical expressions for the photoacoustic time traces and amplitude spectra generated by a sphere with absorbed optical energy only in a spherical shell, and by a sphere with an inwardly decaying optical energy profile. Numerical simulations and experiments were conducted on these two imperfect sphere types. Fitting our model to the simulated or measured spectra allowed us to test our model’s ability to extract the sphere size and optical properties. We found that the radii can be recovered with high accuracy, even when the frequency response of the detector recording the photoacoustic pulse is not precisely known. The model was found to be less sensitive in recovering the optical attenuation coefficient, but it is feasible when the detector’s frequency response is well known.

## 1 Introduction

Photoacoustic (PA) images are usually created based on tomographic principles, where PA time-traces recorded at several detection points are used to reconstruct images of the optical absorbers. Here a higher density and a larger spatial spread of detection points is known to improve the image quality [1], [2], [3]. Acquisition of three dimensional PA images with good imaging quality therefore results in high system costs when high numbers of detectors are used, or relatively long measurement times if the detection aperture is synthesized by scanning of a smaller number of detectors.

The PA behaviour of sources with a simple geometry, like spheres, cylinders and slabs, is well known and is described in detail in literature [4], [5]. From these, we know that the PA signals contain information about the dimensions of the object. Spheres are, for example, known to generate N-shaped time-traces, with a pulse width that is proportional to the sphere diameter, and with a time of arrival that is proportional to the distance between the source and the detector [5], [6]. The amplitude spectrum of such an N-shaped pulse shows an oscillatory behaviour, with peak amplitudes decreasing with frequency and where the peak locations relate to the sphere size [6], [7], [8], [9], [10]. Measuring the PA signal only at a single detection point can therefore allow to locate the PA source in space, while also to recover its 3D geometry, without the need for detector arrays, and reconstruction algorithms.

In the derivation of analytical expressions for the PA response of these well-described targets the assumption is made that the initial pressure is homogeneously generated throughout the entire object, with the object driven with delta pulses to completely comply to thermal and stress confinement [5], [6]. As shown by Hoelen et al. [5], the shapes of the PA time-traces can deviate significantly from the outcomes of the standard models due to use of finite laser pulse lengths and non-uniform pressure profiles within the objects. Since the pressure distribution is proportional to the light fluence distribution within the object for elastically homogeneous targets, Oraevsky et al. [11], [12] used this principle to measure optical properties of material slabs photoacoustically, where the optical attenuation coefficient ($\mu_{eff}$) was derived from rising slope of the PA time signal, the optical absorption coefficient ($\mu_{a}$) from the PA amplitude, and the reduced scattering coefficient ($\mu_{s}^{\prime }$) from a combination of the two. This gave good results in slabs, and should potentially also be applicable in targets with other geometries.

A model describing the PA response of spherical targets with a non-uniform initial pressure profile can potentially find use in various applications. In the biomedical field, at the nano- or micron-scale, one could think of localizing and characterizing exogenous PA or ultrasound (US) contrast or therapeutic agents such as microdroplets [9], [13], microparticles [14] or nanostructures [9], [13], [15] which possess optically absorbing cores or shells. One can also think about macro-scale applications in for example tumour treatment monitoring. Tumour masses can exhibit non-uniform intrinsic optical absorption profiles due to non-uniform vascular distributions. Some tumours types are known exhibit a necrotic core encapsulated by a viable vessel rich rim [16], [17], [18], while smaller or other tumour types demonstrate less necrosis and have vessel growth throughout the entire tumour [19]. Solid and rim like appearances have also been observed in mice studies with PA contrast agents to monitor drug delivery or treatment progression,[20] where tumours presented high PA signal at the rim shortly after injection, but the intensity in the core increased after 24 h.

In this work, we make the first steps in the development of a model that describes the PA response of spheres with a non-uniform initial pressure distribution. Analytical expressions for the PA time-traces of spherical shells and optically homogeneous spheres with an inward decaying initial pressure profile, and the Fourier transforms thereof, were derived. Amplitude spectra were focused on since time-traces from different objects can be difficult to discriminate due distortions in signal shape as a result of detector frequency band limitations [21], while the locations of maxima and minima in the frequency spectrum, that relate to the sphere size, remain unchanged. This feature together with the large number of peaks in the frequency spectrum are expected to enable a robust and accurate determination of the sphere’s dimensions and properties.

The ability of our model to recover the inner and outer radii of the spherical shell, and the radius and the optical attenuation coefficient of the homogeneous beads was investigated with a simulation study in which we tried to fit our models to the spectra from a simulated experiment. To test the model in practice, we measured the PA response of two optically absorbing spherical shells with known radii, and of two absorbing homogeneous spheres with known radii and optical properties. We fitted our model to the measured spectra to recover those parameters. Both the simulations and experiments showed that the model is able to accurately measure the sphere radii. An accurate estimation of the optical attenuation coefficient is more difficult, but simulations show that this is possible when the detector frequency response is known accurately.

## 2 Theory

**Fig. 1:**
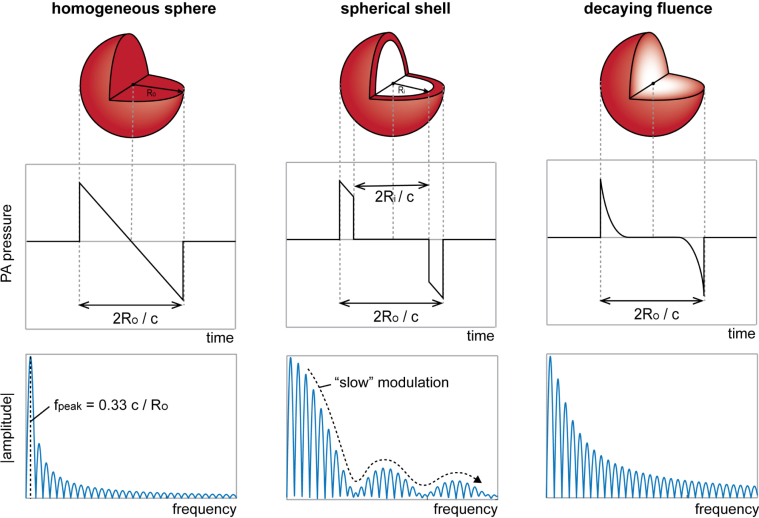
The expected photoacoustic time signals and amplitude spectra generated by spheres with the three initial pressure distributions considered.

To derive expressions for the PA behaviour of spherical shells and spheres with an inwardly decaying pressure profile, we first have to understand the PA behaviour of a homogeneous sphere. The acoustic transient emitted by a spherical object with a homogeneous initial pressure profile recorded at a distance *r* from the centre of the sphere has a typical N-shape [5], [6], [22] as shown in Fig. 1. The width of the time-trace is equal to the time the wave requires to traverse the sphere diameter, the amplitude scales with the absorbed optical energy, and depends on the thermoelastic properties of the object. The amplitude spectrum of an N-shaped pulse shows an oscillatory behaviour, with peak amplitudes decreasing with frequency [8] and with the maximum of the spectrum relating to the sphere size ($R_{o}$) and sound speed in the sphere (c) as $f_{peak}=0.33c/R_{o}$ [7].

The general expression for the PA transient originating from a spherical target yields [22],

(1) $$P\left(r,t\right)=\frac{\mu_{a}\varphi \Gamma R_{o}}{2r}\left(1-\overset{ˆ}{\tau }\right)\Theta_{0,2}\left(\overset{ˆ}{\tau }\right).$$

Where $\mu_{a}$ is the optical absorption coefficient, φ the fluence, Γ the dimensionless Grüneisen coefficient, Θ the Heaviside step function and $\overset{ˆ}{\tau }$ the retarded time defined as:

(2) $$\overset{ˆ}{\tau }=\frac{c}{R_{o}}\left(t-\frac{r-R_{o}}{c}\right).$$

In this expression, it is assumed that the sphere is in free suspension in a surrounding medium with the same density ρ and a speed of sound c. The presence of impedance mismatch with the surrounding medium would modify the time-of-flight for the acoustic transient to reach the detector and create a set of echoes due to the partial reflection at the sphere boundaries.

### 2.1 Spherical shell

The spherical shell model can be described as a sphere with a transparent core of radius $R_{i}$ surrounded by an optically absorbing shell of a certain thickness such that the outer radius is $R_{o}$. The layer’s optical properties are such that the fluence distribution is considered homogeneous within the shell resulting in a uniform initial pressure in the shell. Considering this, we can reasonably assume that the initial pressure field is mathematically equivalent to the difference between two spheres both with homogeneous initial pressure, and radii $R_{o}$ and $R_{i}$ ($R_{i}$ $<R_{o}$)

(3) $$P_{shell}\left(r,t\right)=P_{R_{o}}\left(r,t\right)-P_{R_{i}}\left(r,t\right).$$

(4) $$P_{shell}\left(r,t\right)=\frac{\mu_{a}\varphi \Gamma }{2r}\left[R_{o}\left(1-{\overset{ˆ}{\tau }}_{R_{o}}\right)\Theta_{0,2}\left({\overset{ˆ}{\tau }}_{R_{o}}\right)-R_{i}\left(1-{\overset{ˆ}{\tau }}_{R_{i}}\right)\Theta_{0,2}\left({\overset{ˆ}{\tau }}_{R_{i}}\right)\right]$$

As illustrated in Fig. 1, this transient consists of a positive and a negative peak separated in time, by the time the PA wave requires to travel through the non-absorbing core. The Fourier transform of the time-dependent expression gives the amplitude spectrum of the PA signal coming from a spherical shell as:

(5) P^shell(r,ω)=−A22πω2Bsinω2c(Ro−Ri)cosω2c(Ro+Ri)−α−ωϕRicosωRoce−iωrc

with ω=2πf, $A=\frac{\mu_{a}\varphi \Gamma }{2r}$, $\alpha =\arctan\left(\frac{\omega R_{i}}{c}\right)$, $B=2\sqrt{c^{2}+\omega^{2}R_{i}^{2}}$ and $\phi =\left(\frac{R_{o}}{R_{i}}-1\right)$. The spectrum is the sum of two periodic terms presenting different modulation frequencies: a “slow” modulation in $R_{o}-R_{i}$ combined with a “fast” modulation in $R_{o}+R_{i}$, and an intermediate in $R_{o}$. The spectrum can be predicted to appear as a periodically modulated sine with a global decay in amplitude due to the presence of a pre-factor with inverse dependence on frequency (see also Fig. 1).

### 2.2 Sphere with inward decaying fluence

Here we consider a sphere with radius $R_{o}$ and homogeneous optical properties $\mu_{eff}$ such that there is an inwardly decaying fluence distribution resulting in an inwardly decaying initial pressure distribution. Considering a homogeneous illumination on the sphere surface and the incidence angle of photons at the surface random, each point of the surface of the bead acts as an isotropic point source which can be expressed as: [23]

(6) $$\phi \left(r\right)=\phi_{0}\frac{e^{-\mu_{eff}r}}{4\pi Dr}.$$

Here r is the distance from the source, $\phi_{0}$ the amplitude of the point source, $D=\frac{1}{3\left(\mu_{a}+\mu_{s}^{\prime }\right)}$ and $\mu_{eff}=\sqrt{\frac{\mu_{a}}{D}}$. The total fluence F(r) at a given location inside the sphere is the integration over the sphere surface

(7) $$F\left(r\right)=\phi_{0}\frac{e^{-\mu_{eff}R_{o}}}{\mu_{eff}R_{o}D}\frac{\sinh\left(\mu_{eff}r\right)}{r}.$$

We know that for instantaneous illumination of an object, the PA pressure signal can be expressed as: [24]

(8) $$p_{\delta }\left(\vec{r},t\right)=\frac{1}{4\pi c^{2}}\frac{\partial }{\partial t}\left[\int \frac{p_{0}\left(\vec{r^{\prime }}\right)}{\left|\vec{r}-\vec{r^{\prime }}\right|}\delta \left(t-\frac{\left|\vec{r}-\vec{r^{\prime }}\right|}{c}\right)d\vec{r^{\prime }}\right]$$

with the initial pressure at location $\vec{r^{\prime }}$

(9) $$p_{0}\left(\vec{r^{\prime }}\right)=\Gamma \mu_{a}F\left(\vec{r^{\prime }}\right).$$

For a compact notation, the time window is restricted to $t\in \left[\frac{r-R_{o}}{c},\frac{r+R_{o}}{c}\right]$. The initial pressure locations contributing at the instant t to the pressure signal at the detector location $\vec{r}=\left(0,0,r\right)$ are forming a spherical cap following $x^{2}+y^{2}+{\left(z-r\right)}^{2}={\left(ct\right)}^{2}$ inside the emitting sphere. Considering the bead surface defined as $x^{2}+y^{2}+z^{2}=R_{o}^{2}$, the spherical cap area weighted with the initial pressure values allows to write

(10) $$p_{\delta }\left(\vec{r},t\right)=\frac{1}{2c^{2}}\Gamma \mu_{a}\phi_{0}\frac{e^{-\mu_{eff}R_{o}}}{\mu_{eff}R_{o}D}\frac{\partial }{\partial t}\left[\int_{0}^{r_{lim}}\frac{\sinh\left(\mu_{eff}L\left(r^{\prime }\right)\right)}{L\left(r^{\prime }\right)}\frac{r^{\prime }}{\sqrt{{\left(ct\right)}^{2}-r^{\prime 2}}}dr^{\prime }\right]$$

with $L{\left(r\right)}^{2}=r^{2}+{\left(ct\right)}^{2}-2r\sqrt{{\left(ct\right)}^{2}-r^{\prime 2}}$ and $r_{lim}$ the maximum radius of the projection of the cap on the *xy*-plane

(11) $$r_{lim}=\frac{\sqrt{4r^{2}{\left(ct\right)}^{2}-{\left[{\left(ct\right)}^{2}+r^{2}-R_{o}^{2}\right]}^{2}}}{2r}$$

Finally the acoustic transient can be expressed as

(12) $$p_{\delta }\left(\vec{r},t\right)=\frac{\Gamma \phi_{0}}{4crR}\left[e^{-\mu_{eff}\left[ct-\left(r-R_{o}\right)\right]}-e^{\mu_{eff}\left[ct-\left(r+R_{o}\right)\right]}\right]$$

The corresponding spectrum, normalized by the prefactor $\frac{\Gamma \phi_{0}}{4crR_{o}}$, can be expressed as

(13) p^exp(r,ω)=−1μeffc+iωe−T(μeffc+iω)−1e−iωr−Roc+1iω−μeffc1−eT(iω−μeffc)e−iωr+Roc

with $T=2R_{o}/c$ corresponding to the pulse total duration.

## 3 Materials and methods

### 3.1 Simulation study

Simulations on spheres with different dimensions and $\mu_{eff}$ were performed to investigate the applicability of our model. With sphere parameters given as input, the generation and propagation of the PA wave was modelled with the k-wave toolbox [25]. Simulations were performed on an isotropic grid with a 40μm spacing leading to a maximum supported frequency of 18.5 MHz. The sound speed and the density were set to 1000 kg/m${}^{3}$ and 1482 m/s respectively throughout the entire grid. As a first test, a perfect situation was modelled to verify the analytical expressions for the amplitude spectra of the two sphere types (Eqs. (5), (13)). The time traces were recorded with an ideal point detector (no bandwidth limitations) that was located at 1.25 cm from the centre of the sphere. Two spheres were considered. The first was a spherical shell with $R_{o}=4.8\;\mathrm{mm}$ and $R_{i}=4.2\;\mathrm{mm}$, and the second a sphere with $R_{o}=4.8\;\mathrm{mm}$. The second sphere was imparted with a $\mu_{eff}=5$ cm^−1^, which results in an initial pressure profile decaying towards the centre of the sphere following Eq. (7).

In practice, the ultrasound detector behaviour is not ideal, with a frequency dependent and band-limited sensitivity. Therefore, for experiments or modelling studies, one has to assume that the ultrasound detector has a non-ideal frequency response function. This function can be known from data sheets provided by the manufacturer or can be obtained from earlier experiments using known characterization methods. We call this the “assumed frequency response”. However, the quality of a detector may have degraded in time, a detector may be used with different electronics than the earlier characterization measurements, or errors may have been made during the characterization measurements. The actual frequency response at the time of the physical or digital experiment, we call the “true detector response”, in contrast to the previously characterized frequency response or the “assumed frequency response”.

**Fig. 2:**
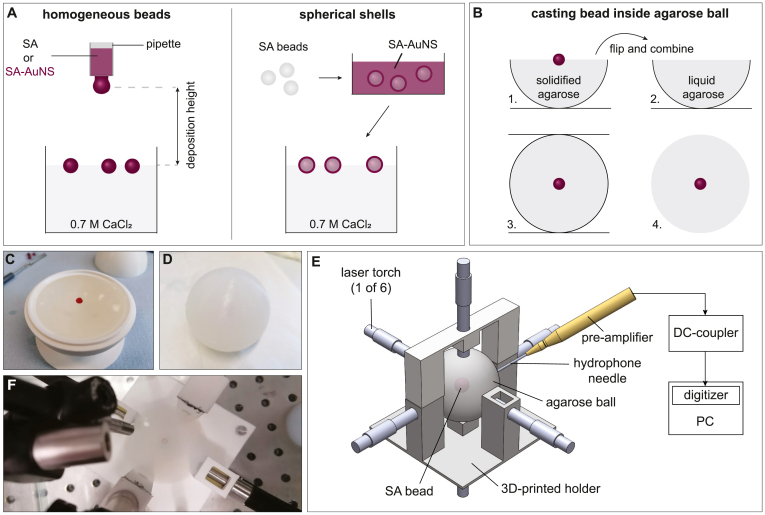
(A) Illustration of the protocol for the production of homogeneous and coated SA beads and for (B) casting them into the agarose ball. (C) Photographs of one of the beads deposited on top of the first agarose hemisphere while the agarose was cooling down. (D) The end result after pouring the second hemisphere on top and removing the ball from the mould. (E) A schematic of the measurement set-up, and (F) a photograph taken from the topside of the measurement set-up. The photograph was taken before the top illumination fibre and the hydrophone needle were installed.

A more realistic set of simulations was performed to investigate the applicability of our model in practice. This true detector response was modelled as a sum of three partially overlapping Gaussians covering a wide band from 0–10 MHz, as in an expected real hydrophone response. Time traces following Eq. (3), (12) were filtered with this true detector response, and random noise with a maximum amplitude of 0.2 times the signal’s standard deviation was added.

To account for the discrepancy between the true and assumed frequency response, we multiplied the analytical spectra with the assumed detector response, and fitted this to the simulated spectra by minimizing the squared error between the two spectra for sphere parameters. The simulated time signals, the true detector responses and the assumed detector responses are shown in Fig. 4. This simulation was performed for 100 different randomly chosen sphere parameters, where $R_{o}$ always had to be larger than $R_{i}$ for the spherical shell and varied between 1 and 10 mm. For the sphere with inward decaying fluence, $\mu_{eff}$ was varied between 0 and 100 cm^−1^, and 1/$\mu_{eff}$ always had to be larger than $R_{o}$/2 to ensure that the pressure decay was not too localized to ensure sufficient frequency content to fall within the bandwidth of the simulated detectors. The true detector response was different for each simulation, with random variations between certain bounds in bandwidth and centre frequencies for the three summed Gaussians. The centre frequencies were varied within 0.5 ± 0.3, 2 ± 1.75 and 5 ± 1 MHz, while their respective bandwidths were varied within 150±30,100±30 and 80 ± 30% respectively. The imperfect knowledge of the detector response used for the fitting was modelled by adding random errors up to ± 10% to the centre frequencies, amplitude and bandwidths of the Gaussians constituting the real detector response.

**Fig. 3:**
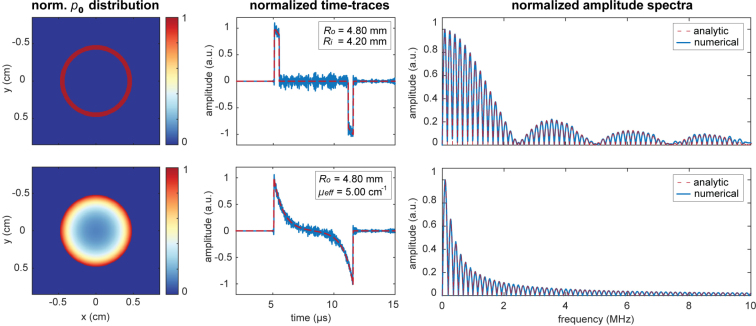
Comparison of analytic expressions with results from k-wave simulations on a spherical shell with $R_{o}$= 4.8 mm and $R_{i}$= 4.2 mm (top row) and a homogeneous sphere with $R_{o}$= 4.8 mm and $\mu_{eff}$= 5 cm^−1^ (bottom row).

### 3.2 Experimental study

#### 3.2.1 Bead fabrication

To verify the applicability of the model in practice, PA measurements were performed on two sets of beads. The first set contained two spherical shell-like beads, each having a different radius and shell thickness. The second set consisted of two homogeneous beads representing spheres with inward decaying fluence, each with a different radius and a different optical absorption coefficient.

The beads were fabricated from sodium alginate (SA) hydrogel (Sigma Aldrich), consisting of the two monomers α-L-guluronic acid (39 v/v%) and β-D-mannuronic acid (61 v/v%) [26]. A 3 w/v% SA solution was prepared and divided into three batches. Gold nanosphere dispersions (AuNS, 13 nm diameter) were also prepared according to the protocol described in Ref. [27]. The AuNS dispersions were added to two of the batches to end with a 2 w/v% and a 1.7 w/v% SA solution corresponding to concentrations of 2.74 $\times \;10^{10}$ and 4.3 $\times \;10^{10}$ particles/ml. No gold nanospheres were added to the third batch. The SA-AuNS mixtures were optically characterized using transmission measurements in a spectrophotometer (Shimadzu, UV2600, Japan). Beads were produced by the drop-by-drop deposition of the mixtures from 8 mm diameter pipettes into a 0.7 M CaCl${}_{2}$ cross-linking solution, from a deposition height of approximately 20 cm (see Fig. 2A). The pipette diameter and the deposition height influenced the bead diameter and sphericity respectively. After around 15 minutes the beads sank to the bottom of the CaCl${}_{2}$ bath, indicating successful gelation.

To create spherical shells, blanco 3 w/v% SA gel beads were first produced and then coated with the absorbing SA-AuNS gel in a subsequent step. The cross-linked blanco beads were immersed in the SA-AuNS solution, assuring total coverage of the bead. Re-deposition of these beads in the CaCl${}_{2}$ bath gelated the SA-AuNS layer around the SA bead. Different shell thicknesses were obtained by varying the immersion time of the bead in the SA-AuNS solution. Fig. 2A illustrates the preparation process.

Several beads were produced of which four beads were selected based on their radii. To centralize a bead inside the measurement set-up, each selected bead was embedded into an agarose ball (see Fig. 2B for a schematic of the protocol). The agarose ball was made by casting an aqueous solution of agarose (3 w/v%), into a spherical mould, which consisted of two 3D printed hemispherical moulds (5 cm diameter) that could be combined. One of the hemispherical moulds was filled with the agarose solution and a bead was positioned on the surface when the agarose had partially solidified. After full solidification, it was flipped and placed on top of the other half sphere which had been filled with the agarose solution, allowing the two halves to stick together. The mould was removed after the solidification of the agarose. After the PA measurements, the SA beads were removed from the agarose and cut into two halves to measure their radii with a microscope.

#### 3.2.2 Experimental setup and measurement procedure

The agarose balls carrying their respective gel beads were positioned centrally in a 3D-printed holder (shown in Fig. 2.E&F). The PA signal generation was achieved by coupling 10 ns 532 nm laser pulses generated by a Quanta-Ray Pro-250 Nd:YAG laser (Spectra Physics, USA) with a 10 Hz repetition rate into a multi-output fibre bundle (CeramOptec GmbH, Germany). The pulse energy was distributed equally over the outputs with an average pulse energy per fibre bundle output of 15 mJ. Considering the 10 ns pulse width and 4 mm fibre diameter, one can calculate that the power density at each fibre output yielded 1.5 MW cm^−2^. The fibre bundles were placed in the holder in direct contact with the agarose ball from 6 sides to aim for homogeneous illumination of the bead. A PVDF hydrophone detector (Precision Acoustics, United Kingdom) with a bandwidth from 0.1–20 MHz was used to detect the acoustic transients from the beads, instead of the typical bandlimited detector used in PA imaging. The reason for this choice was to allow fitting of the amplitude spectra over the broad range since this is a feasibility study of the proposed approach. The active element (1 mm diameter) of the hydrophone was positioned a few mm from the agarose ball surface. The hydrophone was connected to a submersible pre-amplifier (Precision Acoustics, UK) and a DC coupler (Precision Acoustics, UK) followed by a desktop PC with built-in digitizer (DP105, Acqiris, Switzerland) at 500 Ms/s to save the time traces. Averaging was used to increase the signal-to-noise ratio of the recorded signal.

## 4 Results

### 4.1 Simulation study

The unrealistic simulations on two beads using an ideal ultrasound detector have demonstrated that the analytic expressions for the amplitude spectra are correct. Fig. 3 presents the initial pressure distribution slices of the simulated spheres, the recorded time traces and corresponding amplitude spectra. The time traces and amplitude spectra are overlaid with the plots from the analytical expressions (Eqs. (3), (5), (12), (13)) using the known sphere dimensions and/or attenuation coefficient as input. Despite the noise on the time traces, likely originating from the finite grid size in the simulation, a smooth amplitude spectrum is obtained for both cases and they are in good agreement with our analytic spectra.

**Fig. 4:**
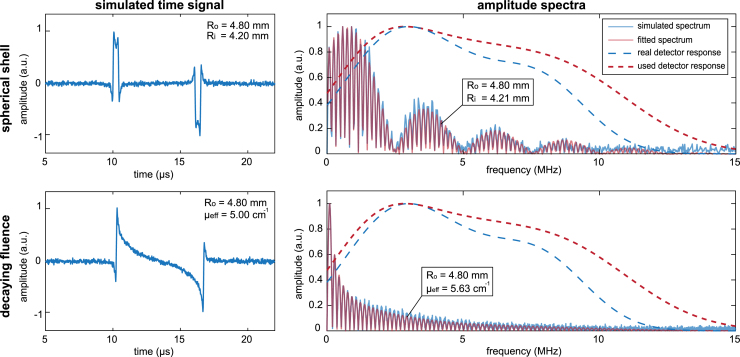
The simulated detected time-traces and amplitude spectra acquired k-wave simulations on a spherical shell ($R_{o}$= 4.8 mm and $R_{i}$= 4.2 mm) and a sphere with an inward decaying initial pressure profile ($R_{o}$= 4.8 mm, $\mu_{eff}$= 5.0 cm^−1^) using an imperfect detector. The amplitude spectra are overlaid with the fitted amplitude spectra.

Fig. 4 shows the simulated time traces and frequency spectra for two out of the 200 simulated experiments. The fitted spectra together with the found radii and/or attenuation coefficient, and the used detector responses are also presented in the Figure. Both for the spherical shell as for the decaying fluence, our model was able to recover the radii very accurately. For both spheres, $R_{o}$ was found to be equal to exactly the set size. $R_{i}$ was slightly overestimated by 0.002% and $\mu_{eff}$ was overestimated by 13%.

**Fig. 5:**
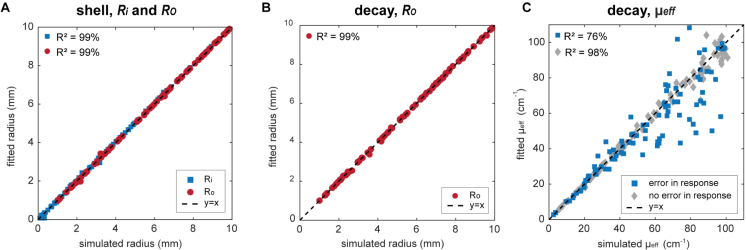
The simulated radii against the fitted radii for (A) 100 spherical shells and (B) 100 spheres with inward decaying fluence. Imperfect detector responses were used in the fitting. (C) A comparison between the accuracy of the model in recovering the $\mu_{eff}$ with perfect and imperfect knowledge of the detector’s frequency response.

Linear regression analysis was performed on the found radii and $\mu_{eff}$ for the 100 spherical shells and 100 spheres with decaying initial pressure profiles (see Fig. 5). R-squared values of 99% for the sphere radii were found while withstanding errors in the knowledge of the detector responses. The model seemed to have more difficulties in recovering $\mu_{eff}$, especially for higher $\mu_{eff}$. An R-squared value of 76% was found. Simulations with a known detector response lifted the R-squared value significantly to 98%. Experiments with a well-calibrated detector could therefore be accurate in recovering $\mu_{eff}$.

### 4.2 Experimental study

**Fig. 6:**
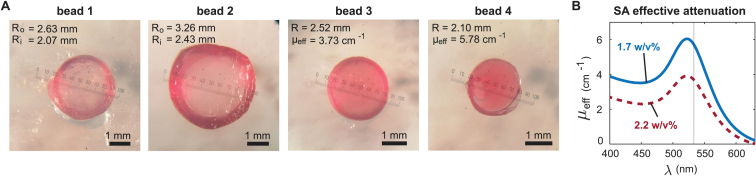
(A) Pictures of the beads. Beads 1 and 2 have a transparent core that is surrounded by an absorbing shell made from the 2.2 w/v% SA solution. The bead radii and attenuation coefficients are shown below the pictures. Bead 3 & 4 are homogeneous beads made from the 2.2 and 1.7 w/v% SA solutions with effective absorption coefficients of 0.37 and 0.58 mm^−1^ at 532 nm respectively. (B) The effective attenuation spectra of the two SA solutions. The gray vertical line highlights the 532 nm excitation wavelength that was used in the experiments.

**Fig. 7:**
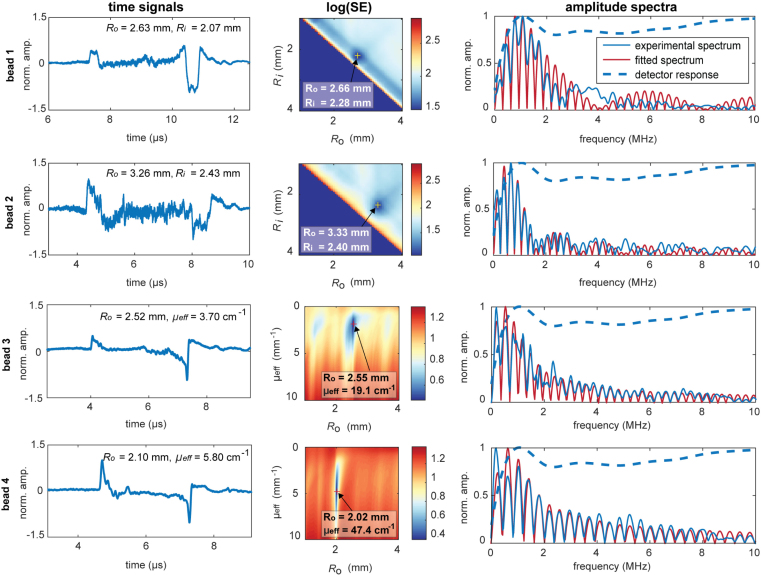
Results from the measurement on the beads. The recorded time-traces generated by the four beads are shown in the left panel. The known bead parameters are written in the figures. The middle panel presents the calculated squared errors (SE) in the range of fitting parameters considered. The minimum SE is highlighted with a cross and the corresponding parameters are written in the inserts. The best fitted amplitude spectrum and the experimentally measured spectrum are plotted in the right panel.

Fig. 6 shows photographs of the selected beads and the absorption spectra of the two SA-AuNS dispersions used in making the beads. The left panel of Fig. 7 shows the time traces of the measurements on the beads. Although the SNR of the signals is limited, two peaks corresponding to the inward and outward propagating PA waves can be observed in all four signals. The signals show some deviations from the theoretical models, (compare with Fig. 4) as a result of band-limited ultrasound detection, internal reflections and imperfect sphericity of the beads. The model did converge for both spherical shells meaning that fitting radii were found. The middle panel of Fig. 7 shows the squared errors between the experimental spectrum and the theoretical spectra for varying radii. The cross cursor highlights the found minimum in each case. The spectra corresponding to the parameters of the found minima are plotted in the right panel of Fig. 7 together with the experimentally measured spectra. For the first 2 MHz of beads 1 and 2, the peak locations in the fitted amplitude spectra match well with the locations of the measured spectrum. For the higher frequencies, the fit becomes less accurate. The radii estimated with our model, are $R_{o}$ = 2.66 mm and $R_{i}$ = 2.28 mm for bead 1, and $R_{o}$ = 3.33 mm and $R_{i}$ = 2.40 mm for bead 2. For both beads, the estimated radii match very well with the sizes observed in the microscopy images which yield 2.63 mm ($R_{o}$) and 2.07 mm ($R_{i}$) for bead 1 and 3.26 mm ($R_{o}$) and 2.43 mm ($R_{i}$) for bead 2.

Fitting our model to the measurements on the homogeneous spheres (beads 3 and 4) was more complicated, as was already realized from the simulation study. The experimental decays can be observed in the two peaks in the time traces which also results in exponentially decaying amplitude spectra. The model was again able to accurately recover the bead radii. Radii of 2.55 mm and 2.02 mm were found for bead 3 and bead 4 respectively, which were measured 2.52 mm and 2.10 mm in the microscopy images. The estimated attenuation coefficients were however highly overestimated. Coefficients of 19.1 cm^−1^ and 47.4 cm^−1^ were found, compared to the ground-truth values of 3.73 cm^−1^ and 5.78 cm^−1^. The vertical smeared-out lines in the squared error figures (middle panel) confirm that the sensitivity of the model in estimating $\mu_{eff}$ is poor compared to the accuracy in radius estimation.

## 5 Discussion

The model as presented in this chapter has a few limitations that should be kept in mind. Firstly, the bandwidth of the generated PA signals should fall within the bandwidth of the detector. When using band-limited detectors, the frequency coverage must be enough to allow identification of either the double modulation in the shell case or single modulation and decay for the homogeneous sphere case. For example, a low-frequency detector used to measure PA signals emitted by a shell structure with a thickness smaller than the main sensitivity wavelength of the detector does not resolve the time trace enough to visualize both modulations in the spectrum. A higher frequency detector with broader absolute coverage may be more appropriate if nothing is known *a priori* about the target.

As also seen in this work, the calibration of detectors needs to be accurate, as the frequency response is implemented into the fitting. In particular, for the homogeneous sphere case, where the slope of the envelope of the spectrum informs on the optical properties, accurate frequency sensitivity estimation is essential.

The radii and $\mu_{eff}$ can only be estimated accurately if the peaks and valleys in the spectra are resolved well. Zero padding of the time traces can suffice, with the consequence of increased computation times. This is usually not a problem for a single target study. But more intensive applications with iterative computation for multiple targets of the corresponding model and detector response can become time-consuming. The signal-to-noise ratio also impacts the quality of the obtained spectra. It can be experimentally improved with simple implementations such as averaging or more efficient delivery of the laser light to the bead or more sensitive detectors.

Then there are also some limitations of the geometrical parameters of the spherical shell. If the considered shell thickness is large compared to the inner core, the proposed approach is prone to failure. In the analytical formulation, the argument of the product of sine and cosine tends to the same value. The double periodicity becomes subtle and in addition to accurate detector calibration, a finer frequency step size is needed to obtain an accurate fit.

In the current definition of the shell model we suppose homogeneous initial pressure in the absorbing layer. This hypothesis holds when the shell is ‘optically-thin’ where $1/\mu_{eff}\gg R_{o}-R_{i}$, meaning that most of the light is transmitted by the shell and contributes to the fluence distribution on the other side of the shell. If $1/\mu_{eff}\gg R_{o}-R_{i}$, the fluence will decay within the shell and the model for homogeneous beads with an inward decaying fluence might be more appropriate to use. Thermal and stress confinement is assumed in our model but is only valid for macro and micro-scaled absorbers excited by nanosecond light pulses. For nano-scale absorbers, the thermal and stress confinement can be violated, meaning that the model loses accuracy. It is known from experimental and theoretical work that PA signals from gold nanoparticles, due to non-conformity of thermal confinement, are caused by heat transfer across the nanoparticle–medium interface and subsequent thermal expansion in the surrounding medium. The temperature dependence of water thermal expansion can provide amplification of the PA signals [28], [29], [30]. According to recent theoretical work [31], the non-linear effects in the proximity of the plasmon resonance peak, can increase the PA signal by more than 50%. This can be an additional contribution to the overestimation in $\mu_{eff}$ observed in our case, but cannot be confirmed in the scope of the present work.

The model can be made more accurate by incorporating more physical properties of the media. Currently, the model assumes an acoustically homogeneous medium and does not account for impedance mismatches that give rise to acoustic reflections between e.g. the sphere and the surrounding medium or the shell and the core of the sphere. In our experiments, hydro-gels in water were used to have minimal impedance mismatches and thereby minimal reflections. In possible future applications, reflections will likely take place which may make the model fitting more challenging. Also, the frequency-dependent acoustic attenuation inside the bead and in the water are not included in the model. High frequencies attenuate more than low frequencies and can thereby complicate the fitting of the $\mu_{e}ff$.

Implementation of more physical properties, like the ones mentioned above, are expected to increase the model’s ability in property determination in experimental situations, but will also make the model more complex. The level of complexity that is required differs per application and is something to be researched in the future.

Experimental imperfections like irregular distributions of optical properties within the shell or sphere, inhomogeneous illumination or imperfect sphericity of the target, could complicate the fitting of our model. Although the SA beads used in our experiment also had imperfect sphericity the model was still able to accurately estimate the radii. This provides the first indications that the model can be robust against imperfections. Further research with simulations on imperfect spheres should point out how these imperfections exactly influence the outcomes and for what imperfections the model is still able to find accurate solutions. Alternatively, some of these imperfections, like the imperfect sphericity could potentially also be implemented in the model in the future.

The presented approach and the feasibility study is meant specifically for measuring the size/optical properties of spherical shaped structures that have been targeted by PA or US contrast agents. These contrast agents extravasate and accumulate either passively or actively at a tumour site. However, the distribution pattern and leakiness of blood vessels in tumours is extremely heterogeneous. Specifically, the microvascular density is high at the invasive edge of the tumour, but sometimes the tumour centre is malperfused, hindering delivery of the contrast agent to the interior region. This is the reason for studying a rim-distribution of absorption (spherical shell), in addition to the homogeneous distribution with realistic radial-decay of fluence.

The method introduced in this work can form the basis of translation to cylindrically-shaped objects. New analytical expressions will have to be derived based on the PA behaviour of cylindrical absorbers. Similar methodologies as proposed can be used, in which case the estimation can also be attempted for cylindrical or line sources and then further on for blood vessels. For those cases, the PA spectrum will be dependent on the orientation of the absorber with respect to the US detector [32]. A caveat is that *in vivo* PA signals will be the integral of multiple sources within the acceptance angle of the detector, so that extraction of the frequency components from specific structures and locations will be challenging.

## 6 Conclusion

In this work, we have proposed an approach to estimate the radii and optical attenuation of spherical objects from a single-point measurement of the PA signals produced. We discuss two cases — that of a sphere with pressure distribution in a shell due to optical absorption at the surface region only, and that of a sphere with inwardly decaying pressure distribution following the fluence profile in an homogeneously absorbing object. We present analytical expressions describing the time traces and amplitude spectra of the PA response of the two cases. The ability of this model in extracting the radii of spherical shells, and radii and optical attenuation coefficients of spheres was first investigated with a numerical experiment where simulations were performed to mimic an experiment. This study showed that the model was able to recover the radii without problems, but that accurate knowledge of the detector frequency response is needed to also achieve accurate estimations of the optical attenuation coefficient. As a next step, an experiment was performed on two spherical shells and two homogeneous spheres made from sodium alginate doped with gold nanoparticles. Experiments on those beads showed comparable outcomes to the simulation study. The radii were recovered accurately, but the estimation of the optical attenuation for the homogeneous spheres was inaccurate. In future studies, a broadband detector with a more accurately known detector response should be used to further investigate the ability of the model to assess the optical attenuation coefficient.

## Funding

This work was part of the 10.13039/501100007601European Horizon 2020 PAMMOTH project under grant agreement No 732411, an initiative of the Photonics Public Private Partnership.

## Declaration of competing interest

The authors declare that they have no known competing financial interests or personal relationships that could have appeared to influence the work reported in this paper.

## Data Availability

Data will be made available on request.
